# Commentary: With a little help from my enteric microbial friends

**DOI:** 10.3389/fmicb.2015.01029

**Published:** 2015-09-25

**Authors:** Walter H. Gunzburg, Brian Salmons

**Affiliations:** ^1^Department of Pathobiology, Institute of Virology, University of Veterinary MedicineVienna, Austria; ^2^AustrianovaSingapore, Singapore

**Keywords:** retroviruses, bacteria, MMTV, HIV, infection

In a recent mini-review published in Frontiers in Medicine, Ben Berkhout pointed out that the interactions between co-localizing viruses and commensal bacteria in the gastric tract are important for the survival and transmission of a number of virus families (Berkhout, 2015). These viruses are part of the virome or viral metagenome that constitutes the total collection of viruses found in and on a multicellular organism (Wylie et al., 2012). Moreover, he highlighted that these close interactions between bacteria and viruses that constitute the microbiome are important also because they may provide opportunities for treatment or even prevention of disease through the modulation of these interactions.

The mini-review furnishes a number of examples of interactions between viruses and bacteria in the gut, including the retrovirus mouse mammary tumor virus (MMTV), the archetypal milk borne virus. As mentioned in the review article, MMTV is a member of the Retroviridae that notably also includes the human disease associated members human immunodeficiency virus (HIV) and human T-cell leukemia virus (HTLV), both of which can also be transmitted to infants by breast feeding. Transmission can occur either by infection with free virions or by delivery of infected cells/cell associated virus where the virus is then transferred through virological synapses that are formed between an infected source cell and a susceptible target cell (Komarova and Wodarz, 2013).

Berkhout's mini-review is interesting and to our knowledge is the first time that the cross virus family relevance of interaction between viruses that infect multicellular eukaryotes and commensal bacteria has been highlighted as a possible general phenomenon. However, we feel that some additional information and some novel points can be brought to the table, specifically with respect to retroviruses. Moreover, the situation with respect to bacterial interaction may be more complicated than was presented, at least for retroviruses and possibly also for other members of the virome.

## Protective vs. non protective effect of bacteria

In the last years there have been a few key studies highlighting a role for bacteria in the milk born transmission of HIV. Martin and colleagues showed that commensal lactobacilli, probiotics that are naturally found in human milk, can inhibit HIV-1 even when they have been heat killed (Martín et al., 2010). This lead Gonzales and colleagues to examine breast milk and gut microbiota in African mothers and their infants (González et al., 2013). They showed that women with HIV-1 RNA in their breast milk have an increased diversity of bacteria in their milk and that lactobacilli were present at higher concentrations, a finding that may be of importance since, as mentioned above, lactobacilli may protect against infection. Nevertheless, it should be noted that in this study the bacterial composition of the infants faces was not significantly different regardless of whether the mother was HIV-1 positive or not, indicating that even if the composition of bacteria in the milk was modified in response to HIV-1 infection, this may not influence the gut flora of breast fed children.

In contrast to the above mentioned examples of bacteria protecting against retroviral infection, another study examined the effects of microbial mediated injuries to the intestinal barrier. This disruption in intestinal integrity or leakiness can be measured by the amount of bacterial lipopolysaccharide (LPS) in the plasma arising from the outer membrane of “bad” gram negative bacteria. Infants exposed to HIV-1 but not infected by the virus were compared to those that became infected and it was found that higher pre-infection plasma LPS levels are a good predictor of milk born HIV-1 infection (Kourtis et al., 2013) suggesting that bacterial LPS facilitates infection. These authors propose that any measures that can enhance mucosal integrity and thus the presence of beneficial bacteria (e.g., prebiotic or probiotic such as lactobacilli as mentioned above) could reduce HIV-1 transmission through breast feeding. Moreover, weaning from breast milk (even though it may contain HIV-1) and antibiotic use should be avoided since both result in the harmful disruption of intestinal mucosal integrity. This was echoed in a recent review in which Nwosu and colleagues pointed out that although microbial mediated enhanced permeability is likely involved in the transmission of HIV-1 and HIV-2, it is not the only factor supporting infection and disease progression (Nwosu et al., 2014).

It has long been known that expression of the MMTV envelope is also regulated by LPS, which could be of relevance for later cell-cell transmission of the virus (Sharma et al., 1988). More recently, Kane and colleagues have provided evidence for a different mechanism in which MMTV virions bind LPS and thus concentrate it in the gut, leading to stimulation of the TLR4 receptor. This stimulation leads to the eventual persistence of MMTV due to interleukin-10 production, thereby preventing antiviral responses leading to tolerance (Kane et al., 2011).

MMTV is well-known as a retrovirus that has evolved to coerce, manipulate or even collude with both the host immune system and its interaction with commensal bacteria in the gut. Over 20 years ago, it was discovered that MMTV encodes a superantigen that is functionally, though not structurally, similar to that found in certain bacterial strains. The superantigen is expressed in infected B cells and presented to whole classes of T-cells which release cytokines that cause the amplification of the infected B cells thereby increasing the pool of MMTV infected cells. Thus, this is an example of a virus independently evolving yet another bacterial like mechanism to facilitate its ability to infect its host via the gut (Ross, 2010). However, in contrast to MMTV envelope expression, MMTV superantigen expression necessary for early milk borne infection is not enhanced by LPS (Sharma et al., 1988; Ross, 2010) perhaps due to different promoter usage (Günzburg et al., 1993; Rouault et al., 2007). While HIV apparently does not encode a stand-alone, separate superantigen product, there is some evidence that the gp120 envelope protein of HIV does have an associated superantigen-like activity in addition to its role in attachment and entry into cells (Planque et al., 2012).

## Commensal bacteria and endogenous retroviruses

As discussed by Young and colleagues, antibodies have established roles in controlling intestinal bacteria and neutralizing products that they produce such as LPS. Using immunodeficient mice, this group showed that infectious virus can arise from usually silent endogenous viruses by exposure to commensal bacteria—presumably expression of these endogenous viruses is normally held in check by the immune response (Young et al., 2012). More recently, the same group presented evidence that such commensal bacteria can modulate the expression of both endogenous mouse and human retroviruses (Young et al., 2014). These findings have implications for cell-cell spread of retroviruses as well as raising the possibility of a role of these endogenous retroviruses in aiding or hindering *de novo* infections with other retroviruses or even with other infectious agents.

## MMTV—a human retrovirus?

The mini-review by Dr. Berkhout states that there is no human counterpart to MMTV—but this is currently a highly controversial statement. Indeed a number of independent groups have documented that there may very well be a human counterpart to MMTV (for recent reviews see Salmons and Gunzburg, 2013; Salmons et al., 2014), although admittedly others have failed to find a linkage (Salmons and Gunzburg, 2013). Nevertheless, MMTV is able to infect human cells in cell culture (Indik et al., 2007; Konstantoulas et al., 2015) and, perhaps even more compellingly, recent evidence has been provided that the putative MMTV-like virus may be transmissible in human milk (Johal et al., 2011) and that presence of the virus in the milk is associated with breast cancer (Nartey et al., 2014). Most recently, evidence has also been presented for saliva as another possible route of transmission of the virus for inter-human infection (Mazzanti et al., 2015).

In summary, the mini-review by Dr. Berkhout is intriguing and scratches the surface of this interesting and somewhat neglected topic. Another recent but short review by Wilks and Golovkina proposes that certain commensal microbiota can either provide protection against virus infection or can promote infection. They further subdivide promotion or facilitation of infection by commensal microbiota into direct and indirect effects (Wilks and Golovkina, 2012). Clearly a larger, multidisciplinary review is needed not only on the influence of commensal bacteria on the transmission of retroviruses but also on viruses from the whole human virome.

### Conflict of interest statement

The authors declare that the research was conducted in the absence of any commercial or financial relationships that could be construed as a potential conflict of interest.
